# The Functional Consequences of Mutualistic Network Architecture

**DOI:** 10.1371/journal.pone.0016143

**Published:** 2011-01-25

**Authors:** José M. Gómez, Francisco Perfectti, Pedro Jordano

**Affiliations:** 1 Department of Ecology, University of Granada, Granada, Spain; 2 Department of Genetics, University of Granada, Granada, Spain; 3 Integrative Ecology Group, Estación Biológica de Doñana, Consejo Superior de Investigaciones Científica (CSIC), Sevilla, Spain; University of Hull, United Kingdom

## Abstract

The architecture and properties of many complex networks play a significant role in the functioning of the systems they describe. Recently, complex network theory has been applied to ecological entities, like food webs or mutualistic plant-animal interactions. Unfortunately, we still lack an accurate view of the relationship between the architecture and functioning of ecological networks. In this study we explore this link by building individual-based pollination networks from eight *Erysimum mediohispanicum* (Brassicaceae) populations. In these individual-based networks, each individual plant in a population was considered a node, and was connected by means of undirected links to conspecifics sharing pollinators. The architecture of these unipartite networks was described by means of nestedness, connectivity and transitivity. Network functioning was estimated by quantifying the performance of the population described by each network as the number of per-capita juvenile plants produced per population. We found a consistent relationship between the topology of the networks and their functioning, since variation across populations in the average per-capita production of juvenile plants was positively and significantly related with network nestedness, connectivity and clustering. Subtle changes in the composition of diverse pollinator assemblages can drive major consequences for plant population performance and local persistence through modifications in the structure of the inter-plant pollination networks.

## Introduction

The architecture and properties of many complex networks, such as molecular and metabolic [1], [2], neuronal [3], genetic [4], [5], social [6], and transportation networks [7], [8], play a significant role in the functioning of the systems they describe. For instance, the fixation of single nucleotide mutations, and gene duplications and deletions, are influenced by the architecture of the metabolic network in *Saccharomyces cerevisiae* [1], where genes encoding enzymes with high connectivity and high metabolic flux have higher chances to retain duplicates in yeast evolution. Similary, the coupling between tie strengths and network topology has important consequences for the global stability of networks generated by mobile phone calls [6]. In these networks, properties like functional robustness, optimal transport, or minimal energy cost directly emerge from the network topology.

Recently, complex network theory has been applied to ecological entities, like food webs or mutualistic plant-animal interactions [9]. Ecological networks have a well-defined architecture, since they are more nested than expected by random models [10], [11], [12], they have a higher density of links, a shorter distance between species, and species are more clustered [13], [14], and thus have strong small-world properties [14]. Previous studies have shown that ecological networks are robust to random losses of species, but probably very sensitive to the loss of key mutualists [15], to extinctions of phylogenetically-related species [16], or to invasions by successful exotic species [17], [18]. Unfortunately, despite the fast-paced information gain on ecological network structure, we still lack an unambiguous link between structural properties of these complex interaction networks and their functional consequences for the dynamics of the system.

In contrast to communities (i.e., assemblages of co-occurring species), populations are groups of individuals interconnected by functions like mating, reproduction, social interactions, sharing of mutualistics, common defense against predators, etc. More importantly, at the network level good estimates of the system functioning, like population performance, population dynamics, demography, recruitment, genetic diversity, etc, may be obtained. However, network theory has been seldomly applied to the study of individual interactions within populations [19], [20], [21], and hence there is not yet a clear view of the relationship between the network architecture and the functioning of individuals within populations. In animal-pollinated plants, the pattern of shared pollinators among individual plants in a population to some extent may be translated into a pattern of mating [21]. If most individuals share the same fauna of pollinators, the resulting pattern of pollen transfer within the population would be less structured than if subsets of plants share distinct groups of pollinators. In this latter scenario there are ample possibilities for assortative mating even in systems with generalist pollination systems. The existence of different levels of structure in the mating and pollination networks may drive significant variation across populations in fitness effects, demography, local genetic structure and gene flow.

In this study, we use complex network theory to study the interaction between a plant species and its remarkably diverse assemblage of pollinators to show that some properties of the individual-based mutualistic networks (the interactions among individual plants based on the pattern of pollinator sharing) can pervasively determine the performance of the whole system. We empirically derived the interaction networks of the herb *Erysimum mediohispanicum* (Brassicaceae) individual plants and their pollinators in eight different populations. Afterwards, we checked whether the architecture of the networks was related to main pollinator assemblage descriptors (abundance, diversity and identity). Finally, we tested whether the network architecture did affect the performance of the plant populations, quantified using a very inclusive estimate, number of juveniles recruited per population.

## Results and Discussion

### Architecture of individual-based pollination networks

**Figure 1:**
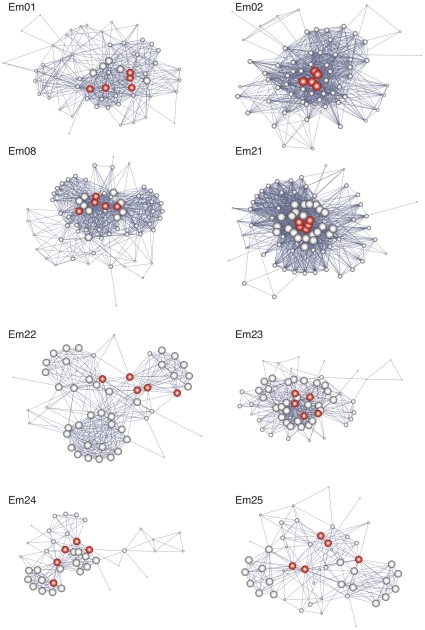
The topology of individual-based ecological networks. Unipartite networks depicting the pattern of shared pollinators by individual plants (nodes) in each population studied (Em01 to Em25). The links among nodes depict the pattern of shared pollinator species (described in Fig. S1); i.e., two nodes are linked whenever they share a pollinator species. The network representation (layout) was generated with the Kamada-Kawai energy-minimization algorithm [29]. Each node represents an individual plant. In green, the most connected plants ( = hubs) in each population. The size of the node refers to the overall flower number displayed by that individual.

We built up intraspecific, individual-based plant networks in eight well-studied plant populations using data on pollinator visitation collected during 2005 (Table S1). We applied network theory tools to the study of these networks, to describe their structural properties [22]. Each individual plant in a population was considered a node, and was connected by means of undirected links to conspecifics sharing pollinators (Fig. 1); i.e., a population-level networks describing how individual plants share pollinators, estimated from the unipartite projections of the bipartite plant-pollinator, two-mode networks (1). In this way, the graph representations not only describe the potential mating events among individuals in each population, but also the specific pollinator species involved in potential pollen transfer. Network architecture was described by means of nestedness, connectivity and transitivity. Collectively, these three measures capture the topology of network architecture. Network nestedness is a measure of the order of the whole system, and quantifies whether the species composition of small assemblages is a proper subset of the species composition of large assemblages. It was calculated using by means of NODF, a nestedness measure based on overlap and decreasing fills [23]. The connectivity of the network measures how individuals are connected to one another through the network [24] and it was described using two parameters: Normalized degree and connectance [22]. Transitivity is a network property that determines the easiness of spread of any factor across the network [24] (e.g., mating events through pollen transfer). Transitivity was estimated by means of the CC1 clustering coefficient, the fraction of connected neighbours around a given individual, an index that measures the local group cohesiveness [25]. All these parameters therefore bear biological meaning in terms of mating and reproductive events in the plant population.

Some individual plants in each population attracted an outstandingly high number of pollinator species, whereas most plants attract a moderate-to-low diversity of pollinators. Consequently, the nestedness values of our intraspecific networks, calculated as NODF, were significant for all plant populations (P<0.0001 in all cases, Table 1). Relative nestedness ranged between 0.065 and 0.137 for Temperature and between 0.015 and 0.898 for NODF (Table 1). Nestedness values were very similar to those found in most interspecific plant-pollinator networks studied to date [10], [12]. Nested networks are organized around a cohesive core of nodes where generalist plant species interact with generalist animal species [10]. High nestedness indicates the occurrence of asymmetric specialization, where the most specialist species interact with the most generalist ones. This is a property that makes the whole system more resistant to the loss of some particular interactions, and favours the persistence of rare, specialist species [10]. The high nestedness of the observed assemblages would consequently explain the high diversity of the *E. mediohispanicum* pollinator fauna, and a highly structured interaction pattern centered on distinct subsets of individual plants in each population that attracted a diverse pollinator assemblage. This may contribute to reduce intraspecific competition and enhance the number of coexisting pollinator species visiting the population [26]. The biological meaning of nestedness in our individual-based networks refers to how pollinator sharing patterns influence mating events among plants. Nestedness implies that individual plants visited by a restricted subset of pollinator species actually share these species with individuals visited by a higher diversity of pollinators. This potentially allows for a more thorough pattern of mating events, with individuals visited by a restricted number of pollinator species not necessarily being reproductively disconnected from the rest of conspecifics. This could increase the probability of successful reproduction and increased population average fitness.

**Table 1:** Among-populations differences in network topology.

Populations	N plants	N pollinators	Nestedness	Relative Nestedness	Normalized Degree	Connectance	Clustering
Em01	63	36	13.08*	0.516	0.225±0.019*	0.221*	0.750 ns
Em02	69	41	15.63*	0.397	0.366±0.024*	0.360*	0.753*
Em08	70	32	16.05*	0.624	0.276±0.020*	0.272*	0.760*
Em21	80	37	22.10*	0.898	0.379±0.024*	0.374*	0.804*
Em22	58	32	11.53*	0.307	0.207±0.0.24*	0.204*	0.703*
Em23	63	39	11.84*	0.294	0.222±0.021*	0.219*	0.773*
Em24	47	30	8.90*	0.115	0.159±0.018*	0.156*	0.650*
Em25	52	32	8.27*	0.015	0.159±0.016*	0.156*	0.697*

Connectance is the number of lines in a simple network, expressed as a proportion of the maximum possible number of lines. Degree is the average number of lines incident with a given node. All network metrics were compared with random-generated networks (see Online Full Methods).

*p<0.0001.

The plants belonging to the same populations were tightly connected among them by sharing many flower visitors. Consequently, our networks exhibited higher connectivity values than expected randomly (P<0.0001 in all cases, Table 1). Network degree, which indicates the average percentage of conspecific plants to which a given plant is connected to, ranged between 15.9% (Em24 and Em25) and 36.7% (Em21). Network connectance, which indicates the proportion of potential inter-plant links that actually occur, ranged between 0.156 (Em24 and Em25) and 0.374 (Em21). These values suggest that the proportion of mating links among plants that were effectively realized was high, up to 37% (Fig. 1). Therefore, individual populations showed ample variation in the degree of pollinator sharing among individual plants, with plants in some populations exhibiting a high overlap of pollinator visitors. Since pollinators mediate the mating system of the individuals by pollen transfer, this means that there exists ample variation among populations in the potential for pollen flow through the population and, presumably, the sizes of individual genetic neighbourhoods.

Despite the high pollinator connection amongst plants from the same population, we found that plants may group in distinct subsets of individuals sharing more similar pollinators. In fact, network clustering was higher than expected by random (P<0.0001, Table 1), ranging from 0.650 (Em24) to 0.804 (Em21) (Table 1). This pattern suggests a high structuring of the individual interaction with pollinators in each population. Variation in this network property indicates ample variation among populations in the potential for assortative mating- i.e., clusters of individual plants that tend to mate among themselves more frequently.

### Effects of pollinator characteristics on network architecture

*E. mediohispanicum* is a very generalist plant visited by over 180 insect species belonging to six orders and as much as nine functional groups (Gómez et al 2007). The structure of the *E. mediohispanicum* pollination networks depended on the abundance, diversity, identity and type of insects visiting the flowers in each locality. Thus, local pollinator richness was significantly and positively associated with network connectivity and clustering (P<0.05, spatial autoregressive models, Table 2), but not with nestedness (P>0.1, Table 2). *E. mediohispanicum* populations with highly diversified pollinator assemblages (Em02, Em21; Fig. 1) characteristically showed more structured patterns of interaction, with a well-defined core of individual plants interacting with a diverse pollinator assemblage that included some generalist insects visiting the subset of more specialized plants. Populations with depauperated pollinator faunas (Em22, Em24, Em25; Fig. 1) lacked a distinct core of generalist plants and showed a less structured network pattern. Local pollinator abundance was also related positively to connectivity (Table 2). These results suggest that an increase in pollinator abundance and, especially, diversity entail a stronger linking among co-occurring plants and a more even mating pattern across the population. In this way we can examine to what extent variation across populations in mean performance relates to local changes in the composition of the pollinator assemblage that translate into variations in the structure of the interaction networks.

**Table 2:** Correlates of pollinator diversity on network topology across the eight *E. mediohispanicum* populations.

	Abundance	S_obs_	Hurlbert's PIE	Bray-Curtis	Morisita-Horn
Nestedness	0.01±0.01	−0.00±0.03	−1.64±0.82 ms	−0.011	−0.049
Degree	0.18±0.05***	0.09±0.03*	−11.67±18.43	0.400*	0.342*
Connectance	0.032±0.01***	0.02±0.01*	−1.61±3.14	0.298	0.332
Clustering	0.11±0.05 ms	0.01±0.002**	−0.83±1.86	0.179	0.235

Figures show coefficients ±1 standard error obtained from spatially-explicit models (for pollinator abundance, S_obs_ and Hurlbert's PIE indices) and partial mantels (method =  spearman), controlling for geographic distance (for Bray-Curtis and Morisita-Horn dissimilitude indices). P-values obtained with 1000 permutations: ms = marginally significant,

*p<0.05,

**p<0.01,

***p<0.001.

**Figure 2:**
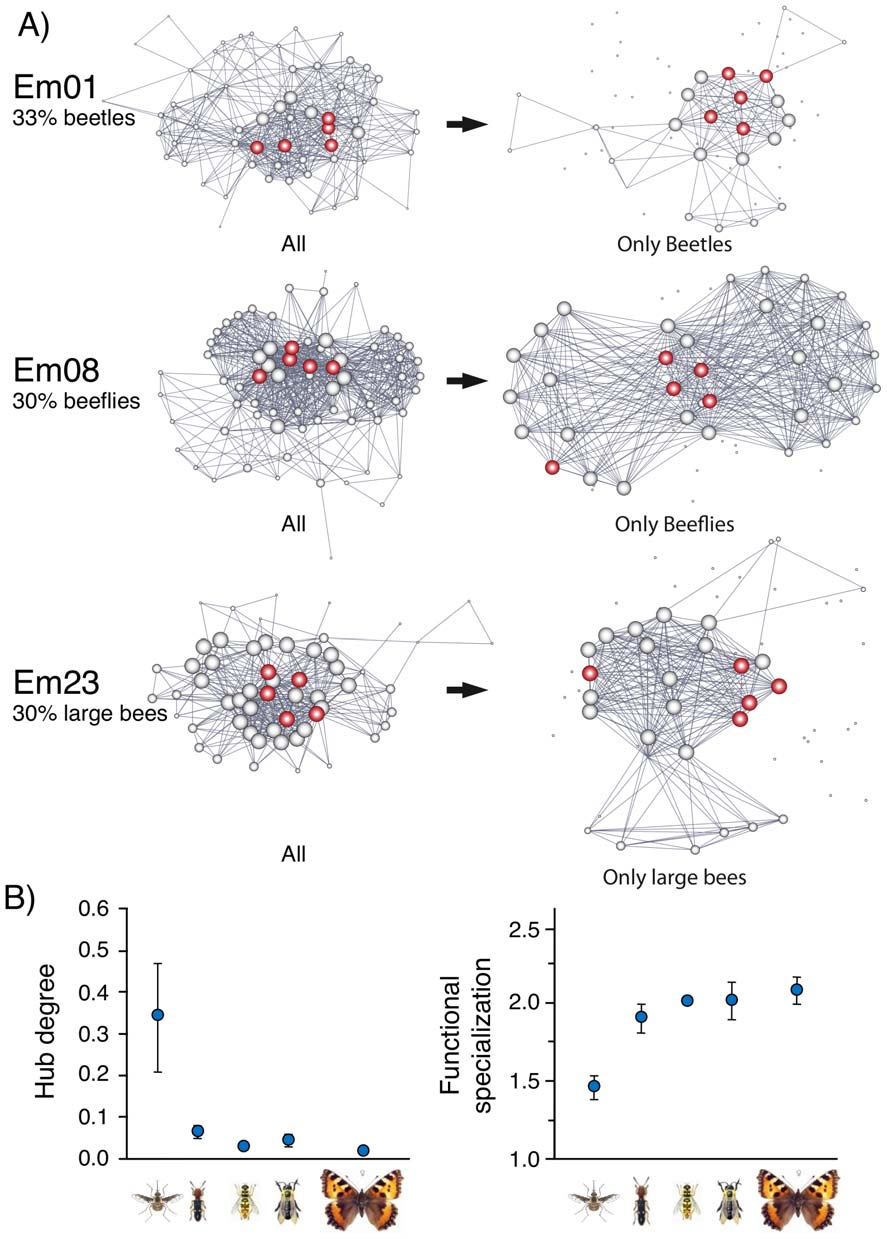
Pollinator effects on network topology. a) Expected changes in network connectivity due to different functional groups of pollinators. Network graphs are depicted for three example populations, illustrating the relationships among individual plants when all pollinator species are included (left) and when only specific subsets are considered (right). Note how the “hub” plants spread all over the partial networks. b) Differences among pollinator type (from left to right, beeflies, beetles, hoverflies, bees and butterflies) in hub degree (F_4,16_ = 21.83, P = 0.0001) and functional specialization (F_4,16_ = 4.13, P = 0.036).

The type of most abundant pollinator in a given plant population determined the connectivity of the local networks (Table 2). Whereas populations with many beeflies had high connectivity (5.319±2.174, t = 2.45, P = 0.050, r^2^ = 0.50), populations with many beetles and hoverflies had low connectivity (−12.016±4.066, t = 2.96, P = 0.050 for beetles; −3.506±1.272, t = 2.76, P = 0.004 for hoverflies). That is, plants in populations where beeflies were abundant were more intensely connected among them, whereas populations with many beetles and hoverflies showed a more defined subdivision of plant individuals in groups with distinct pollinator fauna (Fig. 2a). This is a consequence of the contrasting foraging behaviour displayed by each pollinator type. Beeflies move indiscriminately across the complete set of plants of a given population, whereas the other pollinator groups show a foraging behavior with a high proportion of local movements among close individual plants (i.e., hoverflies) or with very few movements among plants and most movements among flowers of the same plants (i.e., beetles). In fact, both the functional specialization, an estimate of the average topological distance between two given nodes produced by any agent in a network [27], as well as the hub degree, a metric that quantifies the ability of specific agents to connect distant nodes across the network [28], were significantly higher in beeflies than in the other flower visitors (Fig. 2b). These insects have a more central role in the network, potentially mediate pollen flow among a larger number of individual plants when compared to the other pollinator types, and cause more opportunity for mating diversity (Fig. 2b). All these findings indicate that the pattern of pollinator-mediated connections (potential mating events) among co-occurring plants of a given population, and its resulting network architecture, is strongly determined by the type of pollinators visiting the flowers in that population.

### Relationship between plant population performance and network architecture

**Figure 3:**
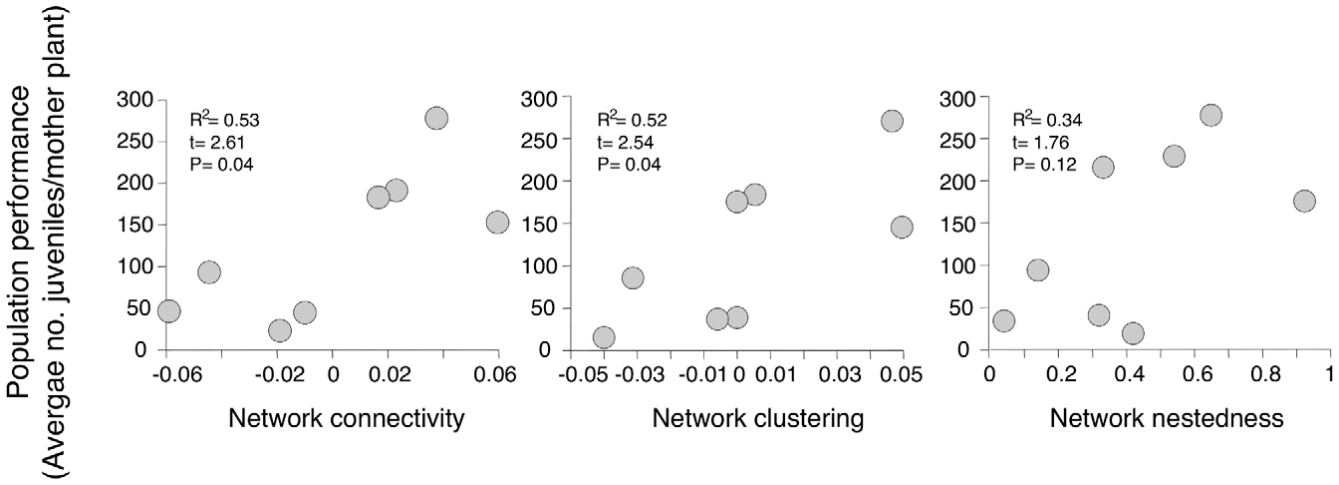
Relationship between network architecture and function. The complex networks of pollinator-mediated interactions among individual plants (e.g., mating events) benefit *E. mediohispanicum* population performance. Populations organized around a core of highly interactive plants (high nestedness) with individuals tightly connected through shared pollinators (high connectivity) within distinct groups exhibiting similar pattern of interactions (high clustering) have high performance (number of juveniles produced per plant).

The topology of our networks had dramatic consequences for the performance of the populations. Variation across populations in the average per-capita production of juvenile plants was positively and significantly related with network nestedness, connectivity and clustering (Fig. 3). Since these analyses were performed after controlling for local pollinator abundance and diversity (see Methods), we can conclude that network architecture itself had a direct effect on population performance independently on any pollinator-mediated effect. In addition, network architecture did not only affect overall plant population performance, but also had significant effects on most intermediate fitness components (Fig. S2–S3). This striking outcome strongly suggests that geographic variation in the local structure of individual plant-pollination networks has a pervasive influence in the outcome of the mutualistic interactions of *E. mediohispanicum* plants in terms of population-level reproductive success. As far as we know, this is one of the first evidences of functional signals of the structural patterns of ecological networks: population variation in plant performance was unequivocally associated with variation in the way that interactions with pollinators were organized. Specifically, our data suggest that high values of nestedness, connectivity and clustering of interactions with mutualistic pollinators are beneficial to individual plants coexisting in local populations. Since we worked with intraspecific networks, where individuals of one plant species interact with each other through many shared pollinator species, our results indicate that the number of successfully recruited new adults in these populations into the next generation will depend on the way individual plants share pollinators locally during the current generation. Therefore, asymmetrical specialization and the existence of a core of supergeneralist plant individuals visited by a very diverse pollinator fauna composed of both generalist and specialist pollinators seems to be beneficial in terms of population seed production, presumably through a more thorough pattern of mating events serviced by a highly structured pollinator assemblage. In addition, our results also indicate that individual plants locally embedded in more connected networks are those producing more seeds. Highly connected networks are those composed of plants more intensely linked through pollinator sharing. Consequently, enhanced pollinator-mediated connectivity in our intraspecific networks probably results in a high frequency of mating events amongst all members of the population, increased gene flow across the entire population, and a reduction of local-scale population genetic structure. These results suggest that nested and highly connected local pollinator assemblages might result in highly structured networks of mating among individual plants, with potential lasting consequences for patterns of gene flow and genetic structure.

We have previously shown that the diversity of pollinators may affect the performance of the plant populations [29]. However, local pollinator diversity is a variable describing the whole system without explicitly taking into account the interactions of the elements of the system (ie, the individual plants). In the present study, we have found that these interactions are also important to determine the function of the system. This is because a high level of local pollinator diversity may happen when different plants are visited by highly contrasting insect assemblages or, alternatively, when all plants are visited by the same very diverse pollinator assemblage. Using a network approach may help to differentiate between both possibilities because it provides critical additional information on the system.

### Conclusion and Perspectives

In summary, the diversity and specific composition of the local pollinator assemblages had a significant effect on *E. mediohispanicum* population performance through shaping geographic variation in the architecture and topology of the intraspecific pollination networks. Nevertheless, there are some important caveats for an accurate view of the functional values of individual networks. First, since nodes in these networks are individual plants, their spatial location has to be included in the analyses. Positional effects can be important in determining specific patterns of interaction with certain pollinator groups. Second, while presence-absence of interactions can provide a broad view of interaction patterns, more robust estimates can be obtained with quantified observations, despite the increased sampling effort needed. Third, the pattern of shared pollinators revealed by our analysis is just a proxy for inferring potential mating events among the linked individuals. More detailed analyses using genetic markers to infer actual mating events in the population could provide a most interesting supplementary view by linking pollinator sharing patterns with actual mating events. Despite these potential limitations, our study revealed consistent trends across distinct populations unequivocally linking network complexity and local population performance.

Our study adds a new dimension to the definition of pollinator effectiveness, and suggests that pollinators may be effective not only by enhancing individual seed production but also by generating thorough pollen dissemination at the population level via their influences on mating events among individual plants. Plant populations mostly visited by effective pollinators would produce more offspring than populations visited by low effective pollinators, an effect ultimately related to how individual plants build up complex networks of interaction with their mutualists. The fact that the pollinator fauna varies geographically in this system gives rise to geographic mosaics of network patterns, with distinct functional signatures on plant performance through fitness effects, as revealed by our results. Our results highlight how subtle changes in the composition of diverse pollinator assemblages can drive major consequences for plant population performance and local persistence through modifications in the structure of the inter-plant pollination network.

## Materials and Methods

### Plant labeling

Ninety plants were marked in each of the eight populations (720 plants in total), at the onset of the 2005 flowering period (April) using aluminum tags attached to the base of the flowering stalks. Plants were monitored throughout the entire reproductive season.

### Pollinator abundance

In 2005, we conducted pollinator counts in the eight populations. During peak bloom (10–15 days per population) we conducted 5 to 7 pollinator surveys per population. In these surveys we noted the number of open flowers in each labelled plant, and the number and identity of pollinator species that landed on the flowers during five-minute intervals. Thus, each survey lasted 450 minutes, and we conducted more than 1500 minutes of observation per population and year (Table S1). Pollinators were identified in the field, and some specimens were captured for further identification in the laboratory. Some rare pollinators could not be captured and thus we only identified them to genus or family [29]. The number of samples per population was fitted to the local abundance of pollinators by means of accumulation curves generated with EstimateS software (http://purl.oclc.org/estimates) [30].

We grouped the insects visiting *E. mediohispanicum* flowers in functional groups. We define “functional group” as those flower visitors that interact with the flowers in a similar manner. Basically, we used criteria of similarity in size, proboscis length, foraging behaviour and feeding habits. Thus, taxonomically related species were sometimes placed in different functional groups. We established eight functional groups: 1) Large bees: mostly pollen- and nectar-collecting females ≥10 mm in body length; 2) Small bees: mostly pollen- and nectar-collecting females <10 mm; 3) Wasps: aculeate wasps, large parasitic wasps and cleptoparasitic bees collecting only nectar; 4) Beeflies: long-tongued nectar-collecting Bombyliidae; 5) Hover-flies: nectar- and pollen-collecting Syrphidae and short-tongued Bombyliidae; 6) Beetles: including species collecting nectar and/or pollen; 7) Butterflies: mostly Rhopalocera, all nectar collectors; 8) Others: nectar-collecting ants, small flies, small parasitic wasps, bugs, and grasshoppers.

### Population performance

Reproductive success was calculated as the number of flowering adults produced per plant at the end of the life cycle, a very inclusive estimate. For this, we calculated per plant several consecutive fitness components. First, we determined the number of seeds produced per fruit by accounting in five fruits per plant the proportion of ovules setting seeds (SO ratio). Second, we estimated the number of seeds produced per plant during its entire life (*E. mediohispanicum* is monocarpic reproducing only once), by counting the number of ripe fruits per plant and multiplying number of fruits/plant by number of seeds/fruit. Third, we quantified seed germination and emergence by collecting 30–40 seeds per plant from each of the surviving individuals per population (N = 335 plants) at the end of the season, when seeds are mature but prior to dispersal (September). We planted 10 seeds per maternal plant on October 2005 in a greenhouse of the University of Granada (UGR). Seeds were placed in individual pots 15 cm apart to avoid competition. To avoid environmental covariance, pots were distributed according to a completely randomized design. We registered seedling emergence during the first month after planting, until no new seedlings emerged. Four, we quantified seedling survival. In order to do this, seedlings were transferred to an UGR outdoor–common garden when they had produced the cotyledons but before true leaf development. Plants were watered once weekly during winter (October–January), twice weekly during spring (February–May) and daily during summer (June–September). The watering regime was identical to all plants. We censused these plants until they flowered in April–May 2007 when they were two years olds. In total, 1675 plants belonging to 332 families reached adulthood.

### Network analysis using graph theory

A network or graph *G*  =  (*V*, *E*) formally consists of a set of vertices *V* and a set of edges *E* between them [28]. For each plant population, we constructed a bipartite network of interacting plant individuals and pollinator species. We only considered plants censused for more than 15 min and receiving at least one visit. Consequently, we studied a different number of plants per population although our initial number of plants was 90 per population, (see Table 1). Afterwards, we obtained the unipartite projections of each bipartite network, depicting the pattern of shared pollinator species among individual plants in each population. We do not consider loops, but allow for multiple lines between two nodes when they share more than one pollinator species. Networks were analyzed with the software package Pajek [31]. We used three main metrics to describe the networks: nestedness, connectivity and clustering.

Network nestedness is a measure of pattern in an ecological system, referring to the order that emanates from the way elements of a particular set are linked to elements of a second set [32]. The more “nested” a system is the more organised it becomes. We have calculated Nestedness using the recently proposed NODF index [34]. NODF index is strongly recommended due its theoretical consistence and its statistical behavior [34]. Since our networks varied in size and connectance, we calculated relative nestedness for NODF [10]. For this, we calculated the NODF using the CE null model. This model considers the probability of a cell a*_ij_* show a presence is
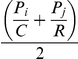
where *P_i_* is the number of presences in the row *i*, *P_j_* is the number of presences in the column *i*, *C* is the number of columns and *R* is the number of rows [33].

Network connectivity indicates link density among the vertices of the network, being an important measure of network robustness and cohesion. Connectivity was estimated by means of two metrics: mean degree, and connectance. The neighbourhood *N_i_* for a vertex *v_i_* is defined as its immediately connected neighbour, as follows:
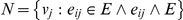

*e_ij_* being the links between the vertices *i* and *j*. The degree *k_i_* of a vertex is defined as the number of vertices, |*N_i_*|, in its neighbourhood *N_i_*. The network mean degree was obtained as the average of the degree for each vertex:
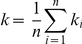



Connectance is defined as the actual proportion of links in a simple network with respect to the maximum possible number of links between all the vertices.

Network clustering was estimated by means of the clustering coefficient. The clustering coefficient *C_i_* for a vertex *v_i_* is then given by the proportion of links *e_jk_* between the vertices within its neighbourhood divided by the number of links that could possibly exist between them. An undirected graph has the property that *e_ij_* and *e_ji_* are considered identical. Therefore, if a vertex *v_i_* has *k_i_* neighbours, *k_i_*(*k_i_* − 1)/2 edges could exist among the vertices within the neighbourhood. Thus, the clustering coefficient for undirected graphs can be defined as
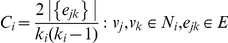



These measures are 1 if every neighbour connected to *v_i_* is also connected to every other vertex within the neighbourhood, and 0 if no vertex that is connected to *v_i_* connects to any other vertex that is connected to *v_i_*. The clustering coefficient for the whole system is the average of the clustering coefficient for each vertex [25]:
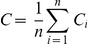



The connectivity estimators were highly correlated (r>0.95, p<0.0001 in all cases). Network connectivity and clustering, but not nestedness, were dependent on network size (r^2^>0.88, P<0.0005 in all cases, 8 networks).

### Comparing real (empirical) networks with random networks

To test whether the structure of our eight empirical networks differed from that of networks produced by pollinators visiting individual plants at random, we compared the metrics (mean degree, connectance and clustering coefficient) obtained from our empirical networks with those of simulated networks. First, we generated random networks to adequately simulate the pattern of non-choosy pollinator visitation across individual plants. To generate these simulated (random) networks, for each empirical population, we first generate a matrix of 100 000 plants x *pol* pollinators, where *pol* is the real number of pollinator species visiting the empirical population. Each cell of this matrix was randomly filled with “zero” (no pollinator visit) or “one” (pollinator visiting that individual plant) depending on the actual relative abundance of pollinators at the empirical population (ie, pollinator frequency of interaction). Each pollinator frequency of interaction was obtained from pollinator censuses in each empirical population (see above). This matrix was randomly re-sampled to build 1000 matrices of *p* x *pol*, where *p* is the actual number of plants of each empirical population with at least one pollinator visit. From these matrices, we constructed 1000 bipartite networks with the same number of vertices as the empirical network. Afterward, we obtained the unipartite projections from the randomly-generated bipartite networks, and calculated for each one the following metrics: mean degree, connectance and clustering coefficient. This procedure produces a distribution of metric values representing the networks produced by pollinators moving among plants without using any phenotypic clue to choose among them. We compared the metrics of the empirical networks with the cumulative distribution of those coefficients obtained from the random networks. All analyses and scripts were done with Pajek [31] and R [35].

### Functional specialization of pollinators

In order to measure functional specialization in pollination networks, we followed the approach developed by Ref [27]. These authors generated a parameter called Functional specialization index to calculate the linkage distance between pollinator types. This index is inspired by the k-neighbours concept in social science [25], and measures the topological distances from a focal node in one set of nodes, A, to each node of another set, B, in the network. Each of these sets of nodes, A and B, may represent different pollinator types. Hence, the smaller the FS values, the more directly type A species are linked with type B species in the pollinator network, and the less strict is the functional specialization.

A hub in a complex network is any component, which acts as a convergence point allowing the transfer of data packets [24]. The hub degree of the pollinators was obtained from unipartite networks generated for these animals using the same procedure as explained above for plant unipartite networks. From these pollinator networks, we calculated the value of the hub degree for all nodes ( = pollinator species) using Pajek algorithm [25].

### Relationship between network structure and function

The effect of network structure on functioning was quantified by means of spatially-explicit models, since most the variables used in this study were spatially autocorrelated [36]. We performed autoregressive models considering spatial-autocorrelation for both dependent and independent variables (lagged-predictor models or SAR_mix_)[37], [38]:
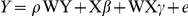



Where ρ is the autoregression parameter, the matrix **W** contains neighbour weights (*w_ij_*) indicating the relationships among spatial units, β is a vector representing the slopes associated with the predictors in the original predictor matrix **X** (network metrics), and γ represents the autoregressive parameters of each of the predictors [37]. In addition, since network metrics were significantly affected by pollinator abundance and diversity, to control for this side effect, we used as a predictor matrix in all these models the residuals of network metrics extracted from a regression including pollinator fauna as independent variable. Therefore, connectance was included in the analysis as the residuals after controlling for pollinator abundance, whereas clustering was the residuals after controlling for pollinator richness. All analyses were performed with SAM [39].

The positive relationship evidenced between network architecture and population performance was not mediated by between-population differences in plant size and flower production, since there was no relationship between number of flowers produced per plant and overall performance (r^2^ = 0.02, t = 0.37, P = 0.72, linear regression).

## Supporting Information

Figure S1 Bipartite networks of each studied population. Circles represent the individual *E. mediohispanicum* plants and squares are the pollinators.
(TIF)

Figure S2 Relationship between network nestedness and the four major estimates of population fitness.
(TIF)

Figure S3 Relationship between network connectivity, measured as connectance, and the four major estimates of population fitness.
(TIF)

Table S1 Location, characteristics, pollinator abundance and diversity and sampling effort of the eight *E. mediohispanicum* populations studied during 2005.
(DOC)

Table S2 Among-populations differences in network topology.
(DOC)

Table S3 Correlates of pollinator diversity on network topology across the eight *E. mediohispanicum* populations.
(DOC)
