# Supplementary figures and images for: The Functional Consequences of Mutualistic Network Architecture
Source: PLoS One. 2011 Jan 25;6(1):e16143. doi: 10.1371/journal.pone.0016143 (PMC3026804; doi:10.1371/journal.pone.0016143)

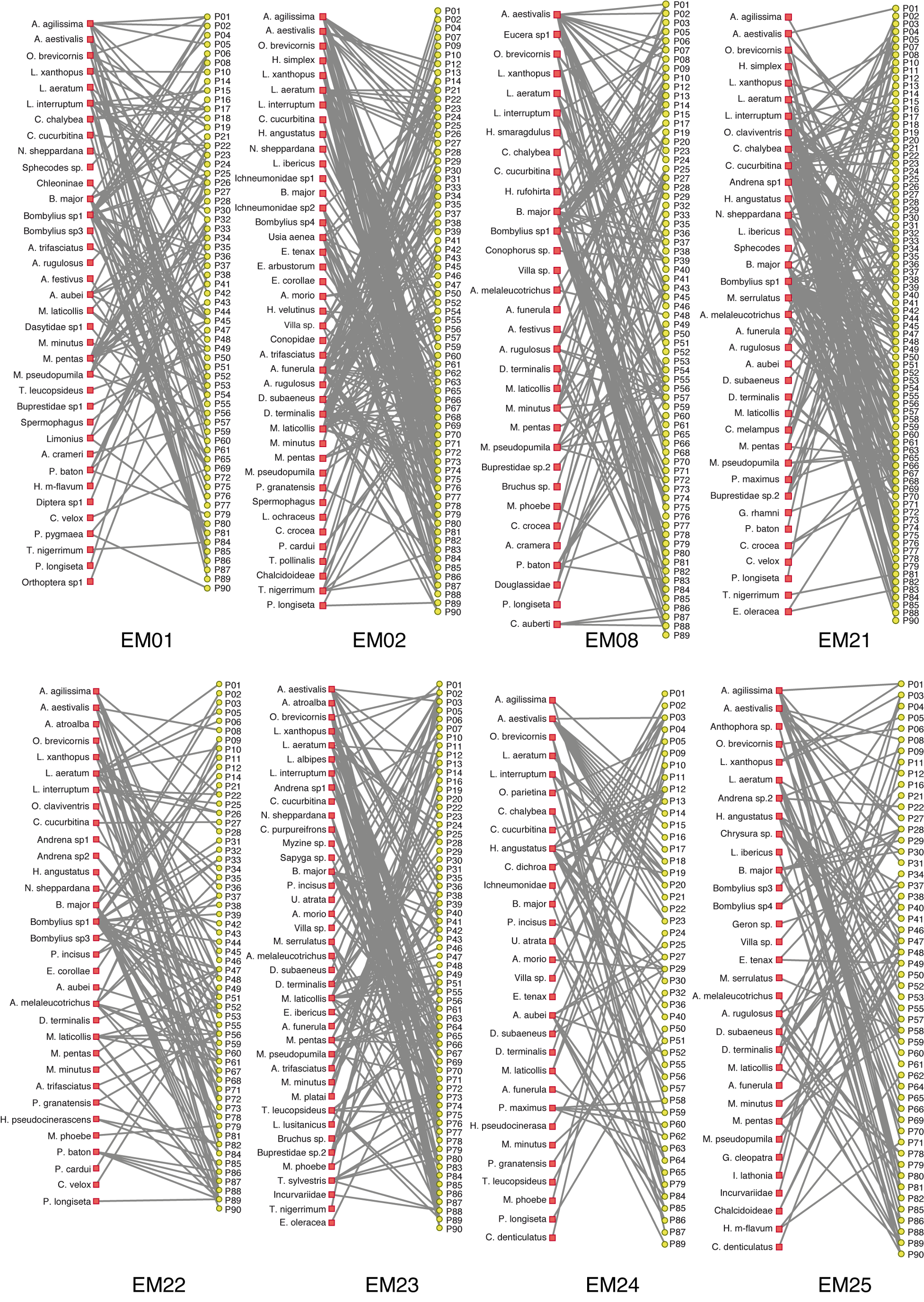

Supplement: Figure S1 — Bipartite networks of each studied population. Circles represent the individual E. mediohispanicum plants and squares are the pollinators. (TIF) [file pone.0016143.s001.tif]

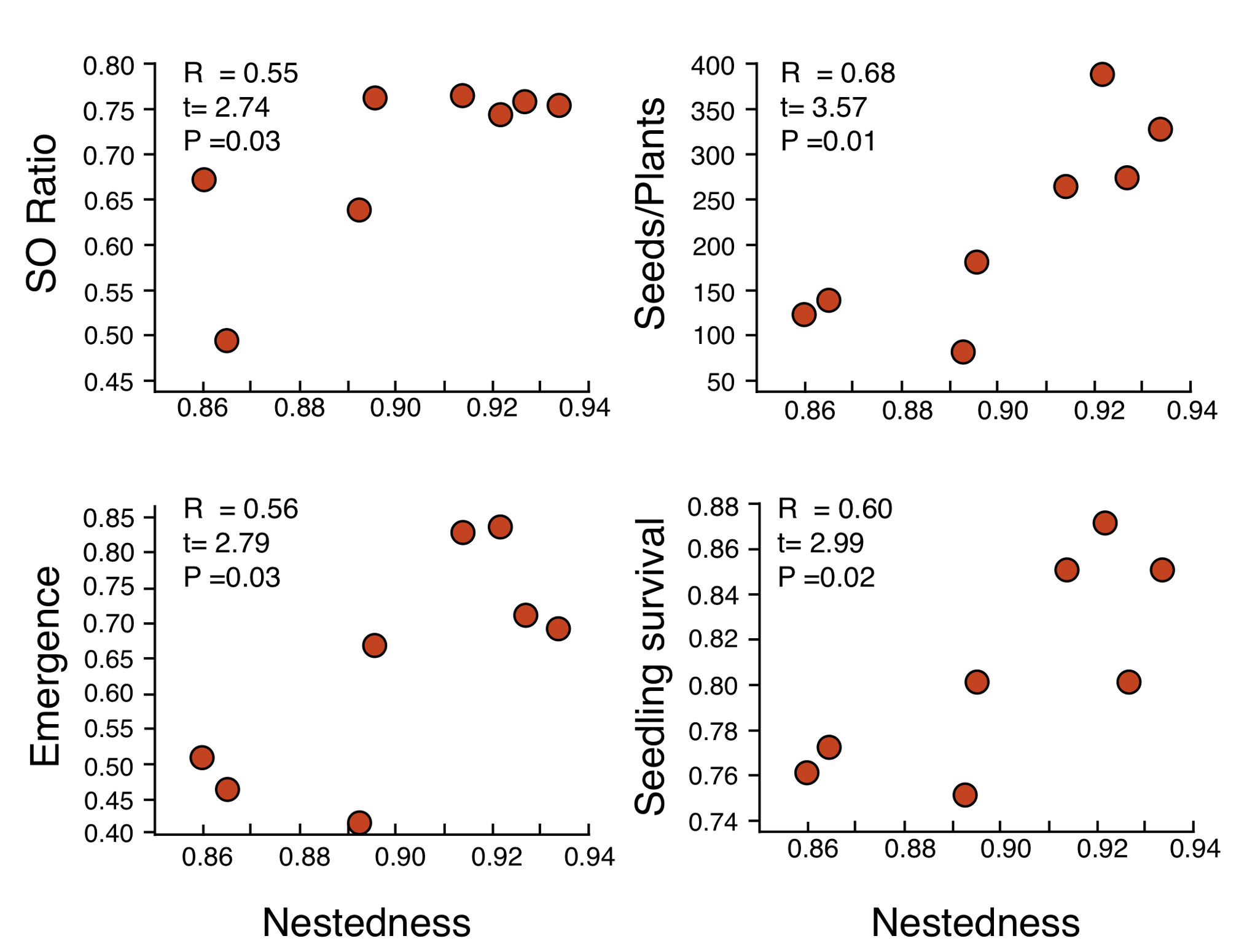

Supplement: Figure S2 — Relationship between network nestedness and the four major estimates of population fitness. (TIF) [file pone.0016143.s002.tif]

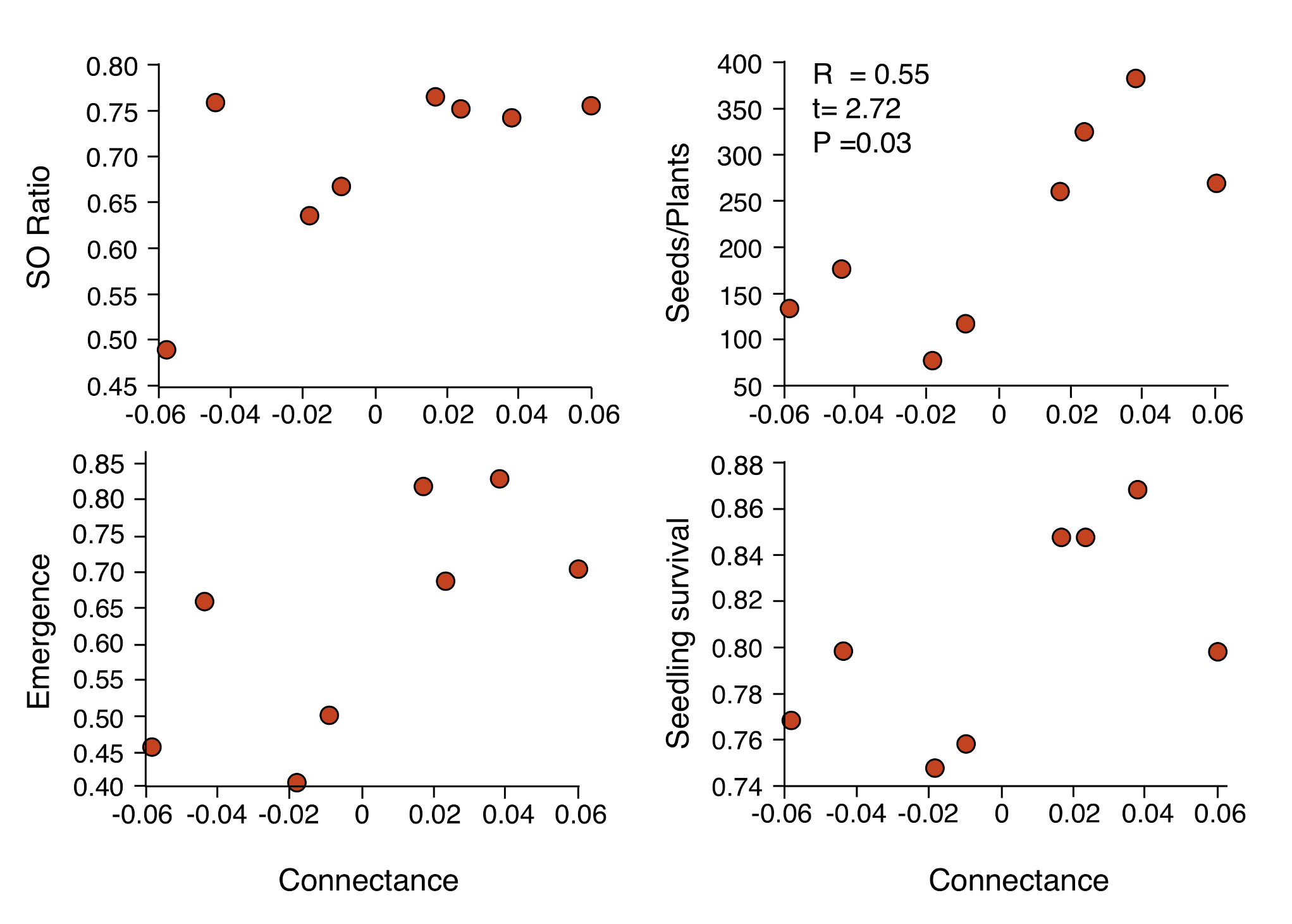

Supplement: Figure S3 — Relationship between network connectivity, measured as connectance, and the four major estimates of population fitness. (TIF) [file pone.0016143.s003.tif]
