# Supplementary material for: The Functional Consequences of Mutualistic Network Architecture
Source: PLoS One. 2011 Jan 25;6(1):e16143. doi: 10.1371/journal.pone.0016143 (PMC3026804; doi:10.1371/journal.pone.0016143)
Supplement: Table S1 — Location, characteristics, pollinator abundance and diversity and sampling effort of the eight E. mediohispanicum populations studied during 2005. (DOC) [file pone.0016143.s004.doc]

**Table S1**. Location, characteristics, pollinator abundance and diversity and sampling effort of the eight *E. mediohispanicum* populations studied during 2005. Abundance (± 1 SE) is expressed as Visits Flower^-1^ Hour^-1^, S_obs_ is the observed number of pollinator species censused per population, S_ICE_ and S_MM_ are estimates of the expected number of pollinator species per population according to the Incidence Coverage Estimate and the Michaelis-Menten Estimate, respectively (Gómez et al. 2007, 2009).

| **Population** | **Latitude** | **Longitude** | **Altitude** | **Habitat** |  | **Abundance** | **S_obs_** | **S_ICE_** | **S_MM_** | **Hurlbert’s PIE** |  | **Plants** | **Minutes** | **Flowers** | **Insects** |
| --- | --- | --- | --- | --- | --- | --- | --- | --- | --- | --- | --- | --- | --- | --- | --- |
| Em01 | 37º 8.00' | 3º 25.69' | 1750 | Forest |  | 0.64±0.07 | 36 | 67.54 | 46.32 | 0.93 |  | 90 | 2395 | 3024 | 162 |
| Em02 | 37º 7.33' | 3º 25.86' | 2099 | Shrubland |  | 1.30±0.12 | 41 | 55.24 | 48.33 | 0.94 |  | 90 | 1720 | 2486 | 270 |
| Em08 | 37º 8.00' | 3º 25.91' | 1690 | Shrubland |  | 0.77±0.07 | 33 | 43.48 | 39.74 | 0.92 |  | 90 | 2085 | 2642 | 169 |
| Em21 | 37º 8.07' | 3º 25.71' | 1723 | Forest |  | 1.60±0.14 | 37 | 49.80 | 43.15 | 0.92 |  | 90 | 1955 | 1826 | 243 |
| Em22 | 37º 7.86' | 3º 25.70' | 1802 | Forest |  | 0.77±0.07 | 32 | 47.69 | 43.09 | 0.93 |  | 90 | 1925 | 1939 | 125 |
| Em23 | 37º 7.74' | 3º 25.58' | 1874 | Shrubland |  | 0.97±0.12 | 39 | 52.08 | 50.40 | 0.93 |  | 90 | 1650 | 2266 | 184 |
| Em24 | 37º 7.51' | 3º 26.14' | 1943 | Forest |  | 0.73±0.10 | 30 | 52.64 | 44.83 | 0.92 |  | 90 | 1485 | 1927 | 117 |
| Em25 | 37º 7.27' | 3º 26.05' | 2064 | Shrubland |  | 0.82±0.10 | 32 | 43.14 | 46.09 | 0.95 |  | 90 | 2195 | 1710 | 118 |
