# Supplementary material for: The Functional Consequences of Mutualistic Network Architecture
Source: PLoS One. 2011 Jan 25;6(1):e16143. doi: 10.1371/journal.pone.0016143 (PMC3026804; doi:10.1371/journal.pone.0016143)
Supplement: Table S2 — Among-populations differences in network topology. (DOC) [file pone.0016143.s005.doc]

**Table S2**. Among-populations differences in network topology. Density is the number of lines in a simple network, expressed as a proportion of the maximum possible number of lines. Degree is the average number of lines incident with a given node. All network metrics were compared with random-generated networks (see Online Full Methods). *p<0.0001

| Populations | N plants | N pollinators | Nestedness (T) | Nestedness (NODF) | Relative Nestedness (T) | Relative Nestedness (NODF) | | Normalized Degree | | Density | | Clustering | |
| --- | --- | --- | --- | --- | --- | --- | --- | --- | --- | --- | --- | --- | --- |
| Em01 | 63 | 36 | 0.9340* | 13.08* | 0.137 | 0.516 | 0.225±0.019* | | 0.221* | | 0.750ns | |  |
| Em02 | 69 | 41 | 0.8928* | 15.63* | 0.114 | 0.397 | 0.366±0.024* | | 0.360* | | 0.753* | |  |
| Em08 | 70 | 32 | 0.9219* | 16.05* | 0.137 | 0.624 | 0.276±0.020* | | 0.272* | | 0.760* | |  |
| Em21 | 80 | 37 | 0.9269* | 22.10* | 0.133 | 0.898 | 0.379±0.024* | | 0.374* | | 0.804* | |  |
| Em22 | 58 | 32 | 0.9142* | 11.53* | 0.133 | 0.307 | 0.207±0.0.24* | | 0.204* | | 0.703* | |  |
| Em23 | 63 | 39 | 0.8651* | 11.84* | 0.065 | 0.294 | 0.222±0.021* | | 0.219* | | 0.773* | |  |
| Em24 | 47 | 30 | 0.8957* | 8.90* | 0.132 | 0.115 | 0.159±0.018* | | 0.156* | | 0.650* | |  |
| Em25 | 52 | 32 | 0.8601* | 8.27* | 0.085 | 0.015 | 0.159±0.016* | | 0.156* | | 0.697* | |  |
