# Supplementary material for: The Functional Consequences of Mutualistic Network Architecture
Source: PLoS One. 2011 Jan 25;6(1):e16143. doi: 10.1371/journal.pone.0016143 (PMC3026804; doi:10.1371/journal.pone.0016143)
Supplement: Table S3 — Correlates of pollinator diversity on network topology across the eight E. mediohispanicum populations. (DOC) [file pone.0016143.s006.doc]

**Table S3**. Correlates of pollinator diversity on network topology across the eight *E. mediohispanicum* populations. Figures show coefficients ± 1 standard error obtained from spatially-explicit models (for pollinator abundance, S_obs_ and Hurlbert’s PIE indices) and partial mantels (method= spearman), controlling for geographic distance (for Bray-Curtis and Morisita-Horn dissimilitude indices). P-values obtained with 1000 permutations: ms=marginally significant, *p<0.05, **p<0.01, ***p<0.001

|  | Abundance | S_obs_ | Hurlbert’s PIE | Bray-Curtis | Morisita-Horn |
| --- | --- | --- | --- | --- | --- |
| Nestedness (T) | 0.01±0.01 | -0.00±0.03 | -1.64±0.82ms | -0.011 | -0.049 |
| Nestedness (WINE) | 0.01±0.35 | 0.01±0.253 | -2.5±-0.49 | 0.323 | 0.255 |
| Degree | 0.18±0.05*** | 0.09±0.03* | -11.67±18.43 | 0.400* | 0.342* |
| Randic Connectivity | 1.68±0.68* | 0.99±0.43ms | -119.29±207.35 | 0.467* | 0.378* |
| Density | 0.032±0.01*** | 0.02±0.01* | -1.61±3.14 | 0.298 | 0.332 |
| Clustering | 0.11±0.05ms | 0.01±0.002** | -0.83±1.86 | 0.179 | 0.235 |
